# The right whale mandatory ship reporting system: a retrospective

**DOI:** 10.7717/peerj.866

**Published:** 2015-03-31

**Authors:** Gregory K. Silber, Jeffrey D. Adams, Michael J. Asaro, Timothy V.N. Cole, Katie S. Moore, Leslie I. Ward-Geiger, Barbara J. Zoodsma

**Affiliations:** 1 Office of Protected Resources, National Marine Fisheries Service, National Oceanic and Atmospheric Administration, Silver Spring, MD, USA; 2 Ocean Associates, Inc., Under Contract to Office of Protected Resources, National Marine Fisheries Service, National Oceanic and Atmospheric Administration, Silver Spring MD, USA; 3 National Marine Fisheries Service, National Oceanic and Atmospheric Administration, Gloucester, MA, USA; 4 National Marine Fisheries Service, National Oceanic and Atmospheric Administration, Woods Hole, MA, USA; 5 United States Coast Guard, Atlantic Area Command, Maritime Security & Law Enforcement Section, Portsmouth, VA, USA; 6 Florida Fish and Wildlife Conservation Commission, Fish and Wildlife Research Institute, St. Petersburg, FL, USA; 7 National Marine Fisheries Service, National Oceanic and Atmospheric Administration, Fernandina Beach, FL, USA

**Keywords:** Endangered whale, US energy imports, North Atlantic right whale, Ship collisions, International Maritime Organization, Shipping industry, Endangered whale, Underwater noise, Economic recession

## Abstract

In 1998, the United States sought and received International Maritime Organization-endorsement of two Mandatory Ship Reporting (MSR) systems designed to improve mariner awareness about averting ship collisions with the endangered North Atlantic right whale (*Eubalaena glacialis*). Vessel collisions are a serious threat to the right whale and the program was among the first formal attempts to reduce this threat. Under the provisions of the MSR, all ships >300 gross tons are required to report their location, speed, and destination to a shore-based station when entering two key right whale habitats: one in waters off New England and one off coastal Georgia and Florida. In return, reporting ships receive an automatically-generated message, delivered directly to the ship’s bridge, that provides information about right whale vulnerability to vessel collisions and actions mariners can take to avoid collisions. The MSR has been in operation continuously from July 1999 to the present. Archived incoming reports provided a 15-plus year history of ship operations in these two locations. We analyzed a total of 26,772 incoming MSR messages logged between July 1999 and December 2013. Most ships that were required to report did so, and compliance rates were generally constant throughout the study period. Self-reported vessel speeds when entering the systems indicated that most ships travelled between 10 and 16 (range = 5–20 +) knots. Ship speeds generally decreased in 2009 to 2013 following implementation of vessel speed restrictions. The number of reports into the southern system remained relatively constant following a steady increase through 2007, but numbers in the northern system decreased annually beginning in 2008. If reporting is indicative of long-term patterns in shipping operations, it reflects noteworthy changes in marine transportation. Observed declines in ship traffic are likely attributable to the 2008–2009 economic recession, the containerized shipping industry making increased use of larger ships that made fewer trips, and diminished oil/gas US imports as previously inaccessible domestic deposits were exploited. Recent declines in shipping activity likely resulted in lowered collision risks for right whales and reduced their exposure to underwater noise from ships.

## Introduction

By the mid-18th century, the North Atlantic right whale (*Eubalaena glacialis*) (hereafter “right whale”) was depleted to near extinction by commercial whaling. Consequently, right whales were among the first of the baleen whales to receive international protection. After whaling for this species ended, attention turned to different threats: serious injury and deaths caused by entanglement in commercial fishing gear and collisions with large ships (Clapham, Young & Brownell, 1999; Kraus et al., 2005). Vessel collisions involving a number of endangered large whale species are relatively common in US waters (Henry et al., 2012; Laist et al., 2001; van der Hoop et al., 2014) and are regarded as a significant impediment to the recovery of right whales (NMFS, 2005). In general, individuals of this species migrate in coastal waters along the US eastern seaboard between feeding/socializing areas in waters off New England and eastern Canada to/from nursery areas off the South Carolina to Florida coasts. The right whale is vulnerable to collisions with vessels throughout its range, but the threat may be particularly high in these aggregation areas where vessel traffic is also concentrated (NMFS, 2005).

As one of the first efforts to reduce the threat of ship collisions with right whales, the United States submitted a proposal to the International Maritime Organization (IMO) in June 1998 to establish two Mandatory Ship Reporting systems (MSR) (USG, 1998). The goal of the MSR is to provide timely information about right whales and their vulnerability to vessel strikes directly to individual vessels as they enter key right whale feeding and nursery habitats.

The MSR proposal was approved by the IMO’s Subcommittee on Navigation in July 1998 and its Marine Safety Commmittee in December 1998 (Silber et al., 2012), becoming the first time an IMO-endorsed measure was used to protect a particular marine species (Johnson, 2004; Luster, 1999) and one of the first formal actions to reduce ship collisions with the right whale. To implement the MSR, the US Coast Guard (USCG) issued a Final Rule in the US Federal Register (USCG, 2001) that codified the systems by amending the US Code of Federal Regulations (33 CFR 169). The US National Oceanic and Atmospheric Administration (NOAA) then added the MSR areas to relevant nautical charts and incorporated the new requirements into various navigational aids such as the US Coast Pilot and elsewhere. As prescribed by the IMO, the two MSR systems became effective in 1 July 1999, and have been in operation continuously since that time. From July 1999 to present, operation and administration of this program have been co-funded and -operated by the USCG and NOAA’s National Marine Fisheries Service (NMFS). All ship-to-shore and shore-to-ship communication costs are borne by these two agencies (including a government contract to the communications provider).

**Figure 1:**
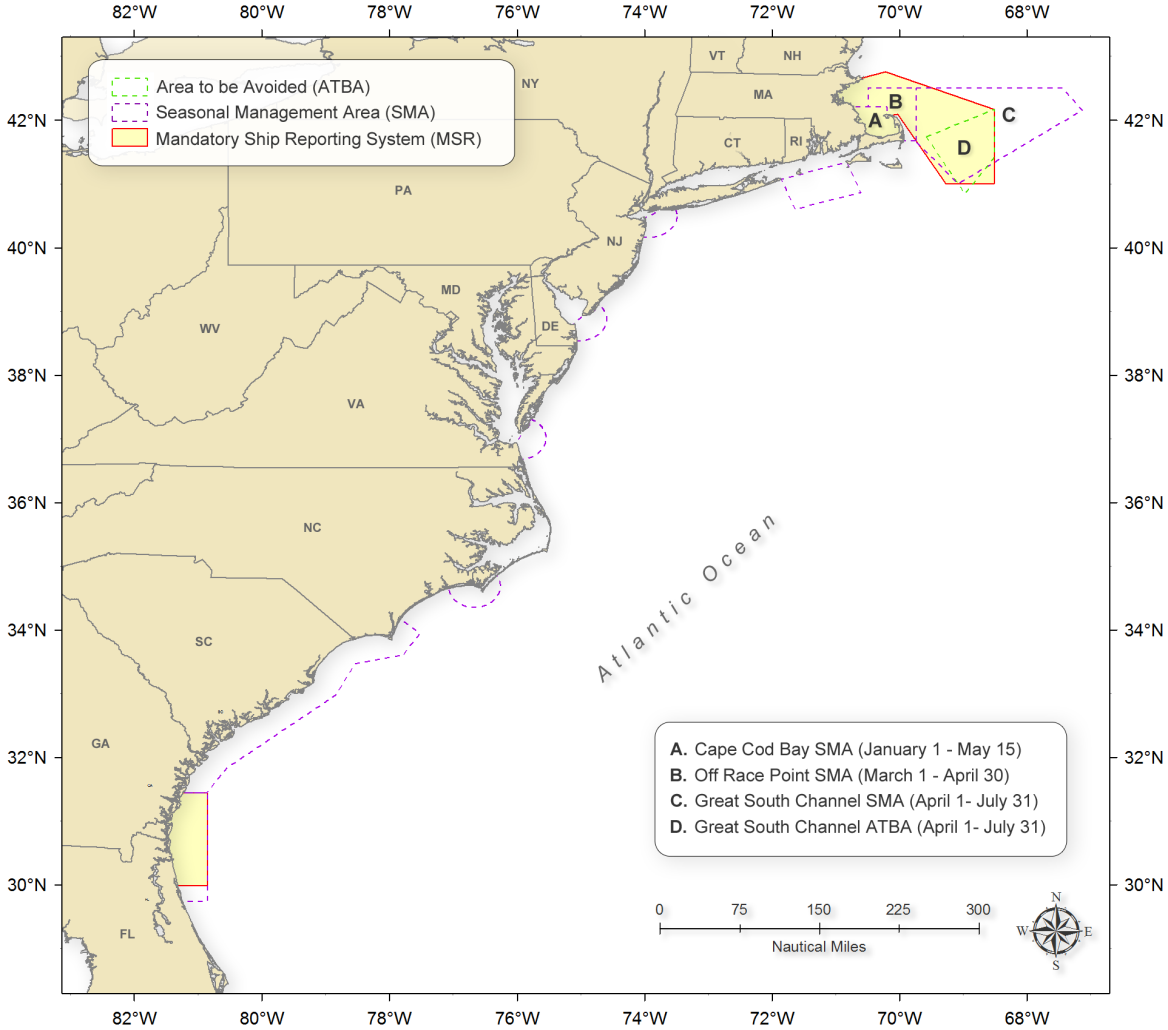
Locations of the Mandatory Ship Reporting systems, Area To Be Avoided, and speed restriction seasonal management areas.

Under the rule, self-propelled commercial ships ≥300 gross tons (gt) are required to report to shore-based stations when they enter either of two regions off the eastern US coast where and when right whales are known to occur: one off the state of Massachusetts operating year-round (a total area of approx. 2,200 km^2^); the other, off the states of Georgia and Florida, is operational annually from 15 November through 15 April (ca. 800 km^2^) (Silber et al., 2012) (Fig. 1). Reports are typically sent as text messages via INMARSAT-C Internet (International Maritime Satellite) and include ship name, course, speed, and destination among other things. Only reporting is required; no other aspect of vessel operations is affected. Incoming reports were parsed and stored on a server for subsequent analysis. An automatically-generated message is returned to the reporting vessel that includes information on locations of recently-sighted right whales; procedural guidance to help prevent vessel/whale collisions; and information concerning additional regulations (e.g., vessel speed restrictions) in place to protect whales from vessel strikes.

The dataset of incoming MSR reports represents a unique long-term record of vessel activities in the areas, in that the system predates similar technologies (e.g., the Automatic Identification System (AIS) and Long-Range Identification and Tracking) that are now used for detailed monitoring of ship locations and operations. When the MSR was established, minimal systematic data regarding vessels entering US ports existed—ships were required only to establish radio contact with the captain of the port at least 24 h prior to arrival. Following the 11 September 2001 terrorist attacks on the United States, systems were developed to more closely monitor vessels making US port calls. Among these, the USCG issued regulations requiring 96-hour notification from vessels (33 CFR 160) (CFR, 2003), known as the Ship Arrival Notification System (SANS). Our study utilized SANS data to assess vessel compliance with the MSR, as discussed below.

Here, we provide a cumulative summary of self-reported vessel activity entering key right whale habitats from July 1999 through December 2013 derived from incoming MSR reports. Our objectives in analyzing and presenting these data, while also recognizing their limitations, were to (a) provide basic summary statistics and a general characterization of vessel traffic in MSR areas since its inception; (b) indicate how trends in vessel activity may reflect changes in various aspects of international shipping operations; and (c) describe how changes in commercial shipping practices may have had unintended implications for right whale conservation.

## Materials and Methods

Mandatory reporting information included the following: system location name (i.e., WHALESNORTH or WHALESSOUTH), vessel name, INMARSAT (satellite communication identification) number, vessel call sign, report date and time, entry date and time, point of entry into these systems, vessel course (heading), vessel speed, destination port, estimated time of arrival at destination, and routing information (e.g., waypoints) (http://www.nmfs.noaa.gov/pr/shipstrike/msr.htm). At a minimum, routing information was provided as a system entry location and a destination. Routing information could also be provided as, or supplemented with, a series of waypoints. Because the data are self-reported and manually entered, these data are not error-free. As such, relevant data fields (entry date and time, entry location, vessel speed, destination port, and routing information) from the MSR reports were vetted before conducting analyses.

### Data quality control

A number of data quality issues were identified when reviewing the incoming reports. Determining whether these issues resulted from misinterpretations of the reporting requirements or data entry issues was beyond the scope of this study. However, a number of actions were taken to address reporting errors. Some reports were sent multiple times; when duplicates were encountered only the most recently logged report was retained. Other reports contained missing entry date and time data or had entry date and time values beyond the temporal bounds of the study period (July 1999 to December 2013). These were removed from our analysis, as were messages that lacked entry latitude and longitude values, had a value of 0 in the entry latitude and/or longitude field, or had invalid entry latitude/longitude values (not between −90 to 90 or −180 to 180, respectively). Reports with entry locations that were more than 5 nautical miles (nm) from a MSR boundary were not included. Some messages contained entry locations that were within 5 nm of a MSR system, but had identified the opposite system in their report (e.g., WHALESNORTH vs. WHALESSOUTH). In these cases, the identified MSR systems for these reports were changed to reflect the system corresponding to the provided entry location. Review of destination port data revealed a number of typographic errors and destination port name variants (e.g., BOSTON, BOSTONPILOT, BOSTOXN, BOSTONMA, BOSTOON). Reasonable efforts were made to resolve these typographic errors and variations.

To test whether transits associated with reported entry locations traveled through the MSR, at least one additional valid location (destination port or waypoint) was required so line features could be created and a spatial overlay performed (this was for validation purposes only and are not presented here). For trips with destination ports located within US waters, Morse Code Alpha (MoA) buoys associated with the port were used as destination locations; the Bureau of Transportation Statistics, National Transportation Atlas Database (NTAD) was used to determine coordinates for destination ports outside the United States. Reports that did not include a valid entry location and at least one additional valid location were removed from our analysis.

Line features were created for those reports containing a valid entry location and at least one additional valid location. For simple transits (those reports that included only an entry location and a destination port), line features were created by connecting a rhumb line from the entry location directly to the destination location. Line features for complex transits (those indicating an entry location and one or more waypoints) were created by connecting rhumb lines between the entry location, provided waypoints, and destination location (when available). For complex transits, initial waypoints that were coincident with the entry location were removed. Similarly, final waypoints coincident to provided destination locations were also removed. A spatial overlay was performed with the resulting transits to determine if they intersected the MSR system associated with their corresponding entry locations. Transits that did not intersect their corresponding MSR system were removed from the analyses. In addition, a visual examination of the transit line features indicated that some mariners submitted reports as they exited MSR systems (reporting that is not required). To remove reports of outbound trips, we included only those with at least 10 nm of travel within its corresponding MSR system.

Operators are also required to report vessel speed when entering the system. Some ships that provided waypoints also indicated speeds for a subset or all of the waypoints; however, for consistency purposes, only the entry speed (as required) was included in our analysis. Transits that met all of the previously described criteria for inclusion, but reported initial speeds ≥50 knots were also excluded.

For the years 1999–2001, compliance requirements with the MSR were documented by the USCG during routine vessel boarding inspections after a vessel arrived in port and were conducted primarily in conjunction with safety and other vessel inspections. In these cases, mariners were asked to provide a ‘hard-copy’ of the return message sent to the vessel via the MSR. Since 2001, the USCG issued 106 civil penalties including warnings and financial penalties to non-compliant mariners. From 2003 to present, SANS data were incorporated into the MSR data base and compliance levels (i.e., each MSR vessel report as directly compared to SANS reports) were computed automatically. Therefore, for compliance rate information reported here, we used only 2003 to present data derived from these SANS-MSR incoming reports comparisons, which we regard as an accurate measure of compliance with the system.

From the vetted incoming reports, we analyzed the spatial and temporal distribution of vessel activity, including: the number of reports, compliance, reported destinations, and vessel entry speeds.

## Results

**Figure 2:**
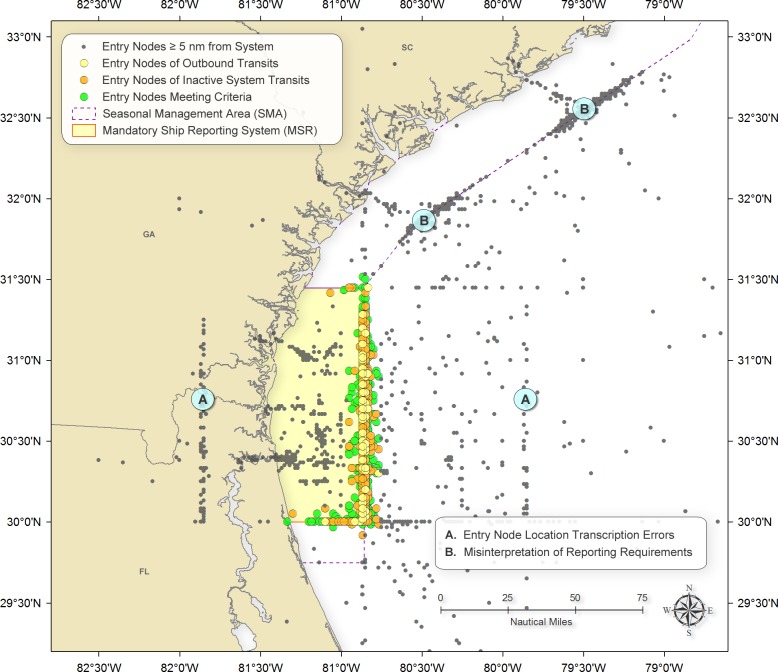
Locations of vessels reporting into WHALESSOUTH. Reports included in the analyses are depicted by green circles. Reports were excluded from analysis if they described outbound trips (yellow circles), were sent outside of the date ranges in which reporting was required (orange squares), or contained starting locations that were >5 nm from the system boundary (blue circles). Of those reports containing starting locations that were >5 nm from the system boundary, many appear to be the result of (A) location data entry errors or (B) reports that were mistakenly provided when entering a North Atlantic Right Whale Vessel Speed Restriction Seasonal Management Areas boundaries (B). Reports used in this study are depicted by green circles. Criteria for selecting these reports are described in Methods.

A total of 46,477 incoming MSR reports were logged between July 1999 and December 2013. Our data quality control eliminated 19,705 of these reports from analysis. We removed 6,505 reports that were either duplicate reports, lacked date information or indicated dates outside of the study period (July 1999 to January 2014), or whose entry latitude and/or longitudes were either missing or invalid (Fig. 2 provides examples of reports with erroneously indicated locations). As noted above, we restricted analyses to reports that included at least one valid location (e.g., a destination or waypoint) other than the entry location, provided an entry location within 5 nm of the MSR boundary, was associated with an currently active MSR area (applies to WHALESSOUTH only), and in which the ship made a trip that passed through the same MSR area as the area indicated in the incoming report. Based on these criteria, 2,091 reports were removed because they did not contain at least one valid location in addition to the entry location; 8,243 were excluded because they did not have entry locations within 5 nm of the closest MSR boundary; and 2,032 were removed because they were reported for WHALESSOUTH when this system was not active. We also eliminated 189 records because the trip did not intersect the MSR area as indicated in the report. Another 487 records were removed because they contained vessel speed values that were not between 0 and 50 knots; and 158 were excluded because they represented outbound transits. In sum, a total of 26,772 reports were used in the subsequent analyses.

Simple operator error appears to be responsible for a relatively large number of incorrectly formatted reports. For example, the grid of reports that surrounds the WHALESSOUTH reporting area, in which locations are offset by one latitude or longitude digit from the reporting area boundary (Fig. 2) suggests that operators inadvertently entered incorrect location information. In addition, a number of reports were submitted by vessels when entering vessel speed seasonal management areas instead of MSR reporting areas (Fig. 2).

**Figure 3:**
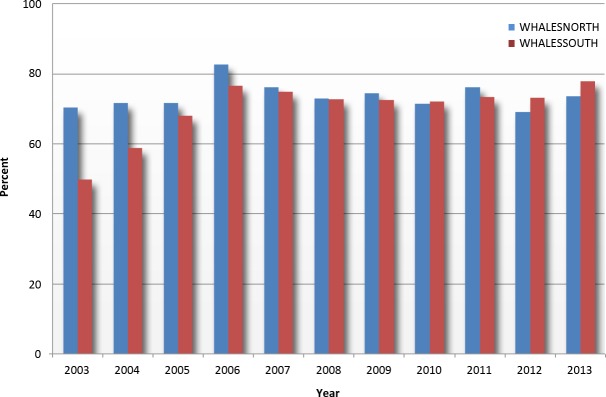
Compliance rates for WHALESNORTH and WHALESSOUTH using MSR reports as compared to USCG SANS data (see text for explanations).

**Figure 4:**
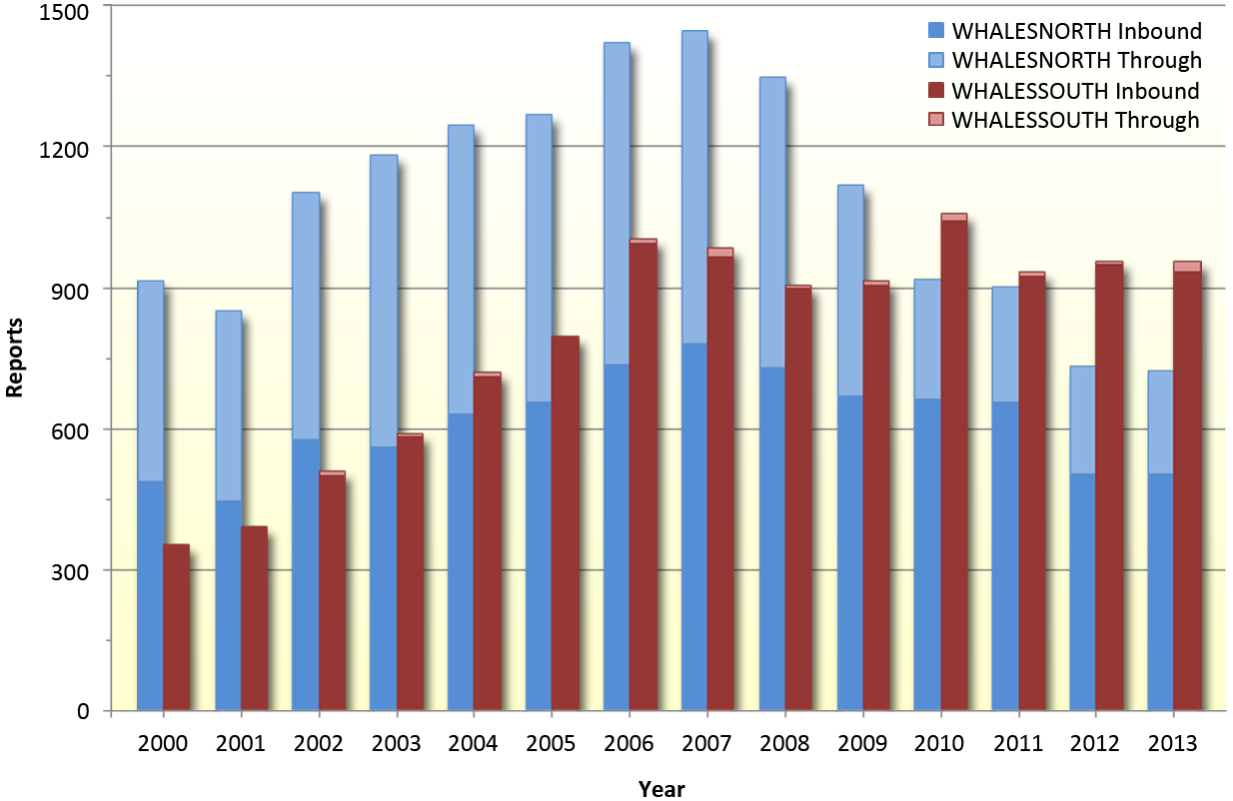
Number of reports into the MSR (meeting data quality criteria described in Methods) for WHALESNORTH and WHALESSOUTH, 1999–2013. Indicated are trips with reported destinations within the reporting area and those that indicated the vessel was traveling through the system to a destination outside the reporting area.

Compliance with the south reporting system was below 70% for a number of years at the outset of our study, but after 2006 compliance in both systems remained generally constant, between 70% and 80%, each year thereafter (Fig. 3). With the exception of years 1999–2002, the total number of vetted incoming reports was greater in the WHALESSOUTH system than in the WHALESNORTH system (Fig. 4). The number of reports for WHALESSOUTH (excluding 1999 which was a partial year of data collection) ranged from 354 (in 2000) to 1,057 (in 2010) and averaged 791 reports per year. In WHALESNORTH, the number of reports (excluding 1999) ranged from 724 (in 2013) to 1,446 (in 2007) and averaged 1,084 reports per year. Monthly counts for WHALESSOUTH averaged 156 reports with a range of 61 (in March 2001) to 216 (in December 2006) (partial months of April and November not included). In WHALESNORTH, the average number of reports monthly was 90 and ranged from 39 (in December 2013) to 174 (in September 2007).

The city of Boston and its associated suite of terminals and ports were listed as the destination for a total of 8,823 vetted reports (Table 1). Jacksonville, Florida and Brunswick, Georgia accounted for a combined total of 9,965 reported destinations in WHALESSOUTH, and constituted the second and third highest, respectively, of all reported destination ports (Table 1). Nearly all ships entering WHALESSOUTH were bound directly for southeast US ports (Table 2), while many vessels entering WHALESNORTH were traversing the area in route to locations outside the reporting area, such as ports in Maine, Canada, mid-Atlantic US states, South and Central America, and the Caribbean (Table 3).

**Table 1:** Number of reports into the MSR (meeting data quality criteria described in Methods) associated with destinations within WHALESNORTH and WHALESOUTH.

Destination	MSR	Reports
Boston and related ports^a^	WHALESNORTH	8823
Jacksonville, FL	WHALESOUTH	7803
Brunswick, GA	WHALESOUTH	2162
Fernandina Beach, FL	WHALESOUTH	835
Kings Bay, GA	WHALESOUTH	258

**Notes.**

a This includes a relatively small number of reports that indicated destinations within or in areas adjacent to the Boston Harbor, including for example, Braintree, Salem, Gloucester, Rockland, Buzzards Bay, Cape Cod Canal, and Provincetown.

**Table 2:** Number of reports into the MSR (meeting data quality criteria described in Methods) associated with destinations outside of WHALESNORTH and WHALESOUTH, summarized by region.

	WHALESNORTH		WHALESSOUTH	
Destination	Reports	% of Total	Reports	% of Total
US Ports	4480	66.4	94	68.3
Canada	1406	20.8	1	0.7
Central/South America	252	3.7	3	2.2
Caribbean	157	2.3	1	0.7
Europe	49	0.7	0	–
Middle East	1	–	0	–
Africa	1	–	0	–
Asia	1	–	0	–
Not reported	396	5.9	39	28.3

**Table 3:** Number of reports into the MSR (meeting data quality criteria described in Methods) associated with domestic destinations outside of WHALESNORTH and WHALESOUTH, summarized by region.

	WHALESNORTH		WHALESSOUTH	
Destination	Reports	% of Total	Reports	% of Total
New England	1882	42.0	0	–
Mid-Atlantic States	1839	41.1	15	16.0
Florida/Georgia	671	15.0	74	78.7
Gulf of Mexico	88	2.0	5	5.3

**Figure 5:**
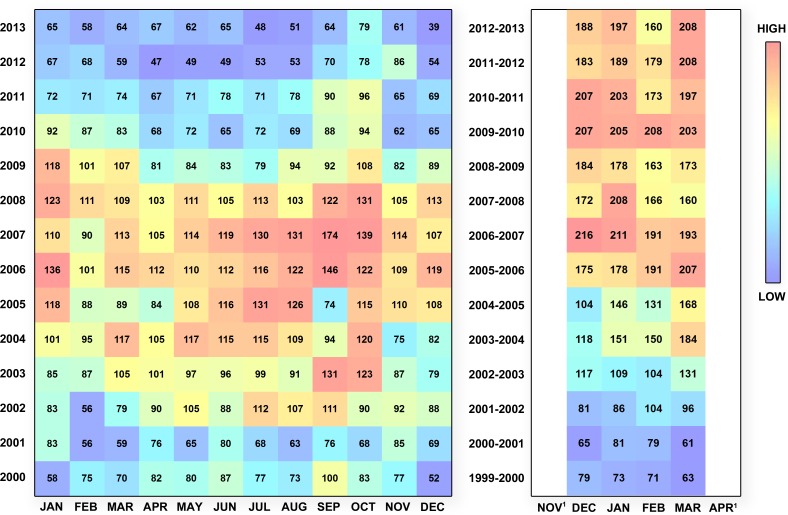
Temporal heatmap depicting the number of reports into the MSR (meeting data quality criteria described in ‘Methods’) for WHALESNORTH and WHALESSOUTH, by month/year.

The number of reports annually into WHALESSOUTH generally increased through 2007 and remained relatively constant thereafter (Fig. 4). Following a steady increase in the number of reports that peaked in 2007, the number of WHALESNORTH reports decreased annually beginning in 2008 (Fig. 4). A 2007–2013 annual decline in reports into WHALESNORTH appears to be driven both by the number of ships bound for Boston and, more strongly, by vessels traversing this system in route to locations outside of the area (Fig. 4). A modest seasonal signal is evident for WHALESNORTH for all years, whereby the number of reports tended to be consistently higher July–October, and in December–January than in other months (Fig. 5).

**Figure 6:**
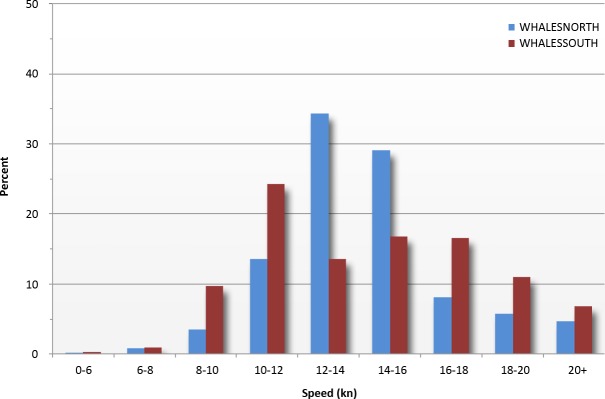
Reported vessel speeds for WHALESNORTH and WHALESSOUTH, 2000–2013.

**Figure 7:**
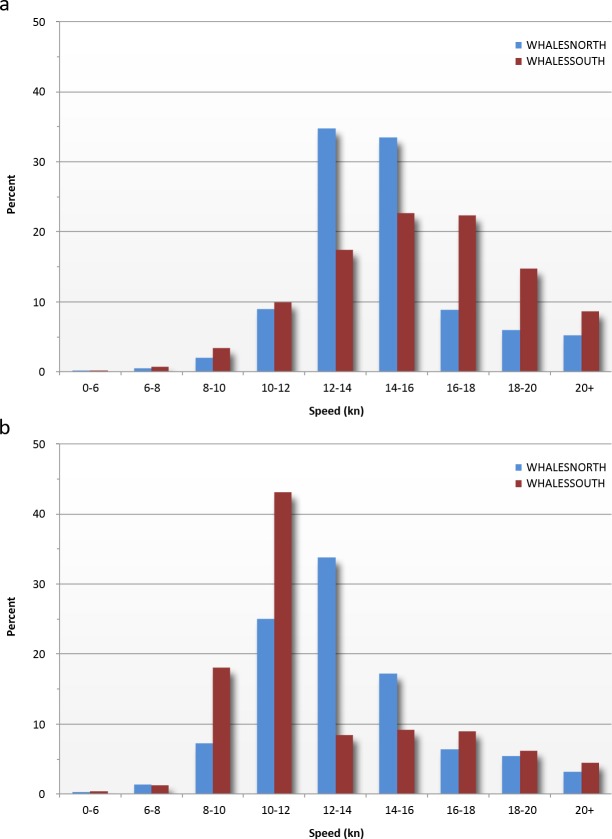
Reported vessel speeds for WHALESNORTH and WHALESSOUTH in (A) 2000–2009 and (B) 2009–2013.

Reported ship speeds ranged from 5 to over 20 knots, with the majority between 10 and 16 knots (Figs. 6 and 7). The distribution of ship speeds reported for WHALESSOUTH differed from WHALESNORTH, with the former appearing to be roughly bimodal around 10–12 and 14–18 knots and the latter more closely approximating a bell-shaped distribution. Reported ship speeds shifted lower in both locations in 2009 to 2013 following implementation of required vessel speed restrictions (Figs. 7A and 7B).

## Discussion

### Value of the MSR

The MSR was established to reduce the threat of vessel collisions with right whales and has provided a means to alert mariners to this threat for over 15 years. While reporting is required for vessels entering the prescribed MSR area, our analysis relies on accurate self-reporting. We did not analyze those records that contained information falling outside the reasonable range of values for the reporting variables. Aside from this, no attempts were made to confirm the veracity of the information contained in the messages; and, prior to the emergence of vessel tracking technologies, there were little or no means to do so. In sum, only about 60% of the total incoming reports were used for the purposes of this study. These limitations notwithstanding, we believe these data represent a reasonable characterization, in relative numbers, of vessel operations in these areas over the 15-year study period.

Regardless of reporting issues or data entry accuracy, reporting vessels received a return message containing right whale conservation information. Thus, the MSR has provided an important function: vessels associated with the over 46,000 incoming reports received messages delivered directly to the ship’s bridge—hundreds of messages each year—regarding right whale vulnerability to collisions by ships.

Reporting compliance rates have been generally constant throughout the 15-year period, with a majority of the required vessels reporting. Due to finite resources and the need to focus on multiple priorities, the USCG has not fined non-reporting vessels since 2006—it has also relied on outreach/education and non-financial civil penalties to encourage compliance. While it is important to enforce any requirement, the MSR program was not intended to be punitive. Instead, the goal of the program was to communicate information and raise awareness.

Clearly, there is some confusion for some mariners about where the MSR reporting areas are located (relative to vessel speed restriction areas, for example) and what is required of them when reporting (Fig. 2). For example, we are told, anecdotally, that ocean-going tug boat operators may report on occasion but not always, when their tug/barge combinations exceed 300gt, but the tug itself does not—representing another example of a lack of clarification about reporting. Improved information and outreach about MSR locations and requirements would improve this situation and would likely enhance the conservation value of the system. In addition, simplifying the reporting format might help reduce transcription errors observed in the data.

### WHALESNORTH vs. WHALESSOUTH

The two reporting areas differed in overall size and the times/duration that they were in effect; and their proximity to various ports had an influence on the character of the trips through each area. When compared to WHALESOUTH, a far greater number of reports for WHALESNORTH indicated trips destined for locations outside the system, including ships bound for various New England ports as well as those that may have been engaged in trade with a variety of US ports, the Caribbean, and European nations (e.g., those inbound for New York from across the Atlantic Ocean) and other trading partner countries (Tables 2 and 3). For these reasons, aggregated vessel operation information for this area (particularly those on voyages through the area) may provide a unique glimpse into patterns regarding commercial shipping activities and international trade, and provide information relative to right whale feeding and socializing aggregations subjected to this ship traffic. It is noteworthy that the overall (and, in some years, the annual) number of reports into WHALESOUTH exceeded those in WHALESNORTH considering the former is operational for only a portion of the year and is smaller in size than the latter, reflecting the importance of the volume of port calls in this region and its implications for key right whale nursery areas. A steady increase (in years 2000–2006) and then a leveling (2007–2013) in the number of reports by vessels entering WHALESSOUTH may reflect the steady or sustained growth of demand for products and services (which includes, for example, popular cruise ship destinations (MARAD, 2012), and containerized, “break bulk” and automobile cargos (Martin Associates, 2013) and the importance of these commodities to the economy of this region and adjoining in-land areas.

Ward-Geiger et al. (2005) provided an analysis of MSR reports, ship tracks, and vessel speeds for July 1999 to June 2002. The distribution of reported ship speeds in our study is generally similar to those presented by these authors, although we found somewhat differing distributions in average speeds in the south versus the north reporting areas. It is not clear why speed distributions differed in the two areas, although adherence with the 2008 vessel speed restrictions (NMFS, 2008) was almost certainly a factor, particularly as compliance with these regulations improved (Silber et al., 2014). Virtually all ships entering the south reporting area were subject to required speed limits after 2008; however, because the north reporting area is not completely concurrent in time and space with speed restriction areas, not all vessels reporting into the north system were subject to the restrictions. Other factors may also contribute to differences in speed distributions, including possible differences in the composition of shipping in the two areas, but assessing additional factors is beyond the scope of our analysis.

### Trends in shipping

Various authors have indicated that the number of large vessels and volume of maritime transport have steadily increased for decades and that continued growth is expected for the foreseeable future (e.g., Corbett, 2004; Dalsøren et al., 2007; Vanderlaan et al., 2009). While these observations may be generally true, they are not borne out in the MSR data, which indicate little or no growth (in the south reporting area) or a decline (in the north) in the number of reporting vessels from 2008 to the end of the study period, presumably reflecting the amount of east coast ship passages and commerce. Thus, the expected trend in traffic volume may be reversing.

The decrease in the number of reports for vessels entering, and in some cases travelling through, the WHALESNORTH area (Figs. 4 and 5) coincides with the “great recession” of 2008–2009 and related global economic dynamics. In 2009, Gross Domestic Product growth dropped 0.6% throughout the world (Labonte, 2010). Between 2008 and 2009, the volume of merchandise trade in the United States declined by 14.0% and 16.4% for exports and imports, respectively; global import and export merchandise trade values likewise fell (UNCTAD, 2013). As global demand for goods declined, the entire supply and delivery chain slowed. The effects of the 2009 recession were largely reversed in 2010 with modest but steady growth in subsequent years, whereby port calls in most locations (MARAD, 2013) and maritime trade and shipping activities returned to or exceeded pre-2007 levels by 2011 (Ex-Im Bank, 2014). The port of Boston was an exception, where imports, as measured by total shipping weight, exhibited a steady decline from a peak in 2004 (NOEP, 2014).

Several factors may be involved in the sustained 2009–2013 annual decline in MSR reports, although determining with certainty the role, if any, of these factors is beyond the scope of this study. The decrease in reports may reflect a continued eroding of commercial shipping activities. However, this seems unlikely as many shipping activities (MARAD, 2013) and most international economies had begun to rebound to pre-recession levels by mid-2010.

Two additional right whale conservation measures, vessel speed restrictions in waters along the US eastern seaboard in December 2008 (NMFS, 2008) and establishment of an Area To Be Avoided (ATBA) in Great South Channel in July 2009 (Silber et al., 2012), may have resulted in modifications of vessel operations or diminished reporting into the MSR. Speed limits may have caused some ship operators to refrain from reporting (perhaps for fear of retribution for reported ship speeds); however, MSR compliance information (as a function of the rather rigorous USCG-SANS 96-hour call-in requirements) (Fig. 3) suggests that the number of reporting ships, relative to those actually making port calls, remained relatively constant after these regulations were established. No strong declines in numbers of trips or vessels in the seasonal management areas were apparent (although modest declines may have occurred in 2012 and 2013) after speed restrictions took effect (Silber et al., 2014), suggesting that operators did not avoid these areas. In addition, we made a cursory examination of vessel AIS data to examine traffic movement in and around the ATBA and found no obvious modifications of routes that would have taken vessels outside MSR reporting areas.

Instead, we believe the MSR reporting data were influenced, at least in part, by significant changes in the composition of the industrial fleet and trade in energy-related commodities. For example, in anticipation of the expansion of a number lock chambers in the Panama Canal, the use of “Post-Panamax” vessels has expanded in recent years. Capable of carrying up to three times the amount of bulk and containerized cargo as most ships currently in use (Rodrigue & Notteboom, 2012), increased use of Post-Panamax vessels is a harbinger of an era in which the transoceanic involves transport of enormous amounts of goods. This will reduce the number of required trips. Post-Panamax ships accounted for 17% of all containership calls at US ports in 2006 and 27% of all US calls in 2011 (MARAD, 2013). From 2006 to 2011, the average vessel size per US port call increased (and the average age of ships in the world’s fleet dropped as new large ships are built and put into service) (DOT, 2013), while calls by smaller vessel classes decreased (UNCTAD, 2013). Therefore, an increased use of large ships may account, at least in part, for the reduction of reports into the MSR. Ports in New York and New Jersey, Baltimore, Maryland, and Mobile, Alabama have already dredged their harbors to accommodate these large ships, while Savannah, Georgia, Charleston, South Carolina, and Jacksonville, Florida either have channel modifications underway or planned for this purpose.

Another large scale shift in US imports/exports took place in this same period. Natural gas and crude oil production in the United States has steadily increased in the last five years with the development of new (primarily shale gas) sites and with increased use of hydraulic fracturing and horizontal drilling technologies (EIA, 2014a; Humphries, 2014). As a result, US natural gas imports have declined annually since 2007; in 2009, for the first time, the country’s domestic gas and oil production outpaced its imports (EIA, 2014b). The Marcellus shale gas field of Pennsylvania and West Virginia, alone, yielded virtually no natural gas in 2007, but is projected to provide nearly one-quarter of the United States’ gas in 2015. As sources and destinations for oil have rapidly evolved (PIRA, 2014) the complexion of water-borne shipment of these commodities has shifted in the areas we studied and elsewhere. As one example, liquefied natural gas (LNG) imports from Trinidad/Tobago (the largest LNG exporter to the United States (EIA, 2014b)) to Everett, Massachusetts (a destination port in our data) declined each year from over 180 million cubic feet in 2007 to 52 million cubic feet in 2013 (EIA, 2014b). In addition, a number of locations, including the ports of Saint John, New Brunswick, Canada (which hosts Canada’s largest crude oil refinery; Tremblay , 2013) and those in Trinidad/Tobago and Venezuela (crude oil) MSR reported destinations for ships passing through WHALESNORTH. Although our data lack sufficient specificity (e.g., vessel type designations) to allow definitive statements regarding shifts in oil/gas trade, we believe the observed 2009–2013 declines in vessel reports reflect the profound, ongoing changes in the transport of these materials.

Considerable variation becomes clear when numbers of reports are considered on a monthly basis (Fig. 5). In some years, certain seasonal fluctuations also appear to be occurring which may reflect influxes of passenger, cruise, and large recreational vessels in summer; or the movement of heating oil or other seasonally-important commodities through WHALESNORTH.

In addition, several months exhibited atypically low numbers of reports relative to months that preceded or followed it—or in the same month in other years. This pattern may be attributed to hurricane activities and other significant large scale events that limited maritime commerce. For example, one of the lightest reporting months (relative to the same month in other years) occurred in September 2005 (Fig. 5) when hurricanes Katrina (making landfall 30 August 2005) and Rita (landfall on 25 September 2005) battered Gulf of Mexico coasts, keeping ships in port or at sea to avoid the storm and slowed or stopped production of Gulf coast oil refinery facilities. August and September 2004 were also relatively light reporting months relative to those same months in other years coincident with four Category 2 or greater hurricanes that struck the Gulf of Mexico, Florida, and mid-Atlantic state coastlines. In contrast, we see no particularly strong signal in the number of reports from hurricane Sandy (October 2012) and other storms that brought destruction on large geographic scales. The Gulf of Mexico’s Deepwater Horizon oil spill beginning in late April 2010, and related activities to rescue lives and contain oil, likely disrupted supply chains and contributed to reduced vessel activities in the Gulf and elsewhere and may account for relatively fewer MSR reporting in May and June of that year relative to the same months in other years.

### Shipping activities and right whales

Regardless of reasons for shifts in composition and evolving practices in international shipping fleets, reductions in the relative amount of ship traffic in the last five years likely resulted in important consequences for right whales and other large whale species. Several authors have reported that the economic downturn of 2008–2009 resulted in reduced ship traffic and, consequently, a corresponding decrease in the amount of oceanic noise as introduced by large ships (Andrew, Howe & Mercer, 2011; McKenna et al., 2012; Miksis-Olds & Bradley, 2013).

And related to this, Rolland et al. (2012) reported that the absence of ships following the terrorist attacks in the United States on 11 September 2001 resulted in less underwater noise and lowered baseline levels of stress-related hormone metabolites (glucocorticoids) in right whales in the Bay of Fundy, Canada. These and other authors noted the strong link between chronic elevations of glucocorticoids and suppressed immune systems, impaired individual health, and population declines in a number of vertebrate populations (e.g., von der Ohe & Servheen, 2002; Romero, 2004). However, unlike the Bay of Fundy study, when comparing (a) two-week periods before and after the 11 September 2001 terrorist attacks, and (b) September 2001 to that month across all years, we found no change in the number of ship reports including those reporting northbound transits into Canadian waters.

More generally, assuming that (a) the MSR data are truly indicative of relative levels of (and declines in) shipping activity in US northeast ports, and (b) these declines are accompanied by a decrease in radiated ship noise in waters in and around New England, the Canadian maritime provinces and perhaps elsewhere throughout range of right whales, then the species may have been exposed to a soundscape and disturbance from noise that the species has not experienced for nearly two decades. Therefore, declines in supply and demand for certain goods may have resulted in increasingly hospitable right whale habitat.

A decrease in the number of ship transits would also suggest that right whales and other large whale species have experienced lowered exposure rates to the potential for fatal collisions with large vessels. Recent decreases in both the number (Laist, Knowlton & Pendleton, 2014) and probability (Conn & Silber, 2013) of fatal ship strikes of right whales has been attributed to the 2008 creation of vessel speed restrictions in right whale habitat. However, a reduction in the actual number of trips in these areas may also have had a role in reducing strikes. Known fatal right whale/vessel collisions occurred at an average rate of 1.0 per year in 1996 to 2001; increased to 1.7 per year 2002 to 2007; and fell to 0.5 per year 2008 to 2013 (MMC, 2014).

## Summary and Conclusions

Submitting a message into the MSR is required for certain vessel classes, but the content and accuracy of these messages rely on “good faith” self-reporting. Numerous reports contained mistakes such as transcription errors. Errors in incoming messages notwithstanding, all reporting ships received a return message; and hundreds of messages were sent to reporting ships each year since the MSR’s inception. For this reason alone—and because it was one of the first formal measures aimed at reducing the threat of ship collisions with right whales—the MSR has probably provided an important function in notifying a broad international community about vessel/whale collisions. Steps should probably be taken to better equip ships’ captains and mates about reporting requirements and to further enhance the overall conservation value of the information provided through the program.

The MSR has also provided a relatively long time series characterization of shipping operations that, in part, pre-dates more rigorous vessel monitoring programs and technologies. We believe the data set of accurately entered and transmitted reports provides a reasonable15-year representation of maritime transportation activities in these areas. Among other things, reported speeds were largely consistent with those determined from remote monitoring programs and reflect the maritime community’s response to additional measures to minimize right whale ship-strike rates.

If the number of incoming reports is truly indicative of shipping practices, a number of economically-driven changes in marine transportation activities, as well as large scale meteorological events, appear to be reflected in the data. Although we are not able to determine their role with certainty, it appears that global and industry-wide events may have had unanticipated benefits in reducing shipping-related impacts to right whales. Among these, a troubled worldwide economy in the late 2000’s and slowed or diminished supply chains that move various commodities such as oil and gas resources likely reduced the overall amount of ship traffic. Industry-wide shifts toward larger ships conveying containerized goods have also altered the complexion of maritime transport during our study period. As a result, right whales may have been exposed to a lowered risk of collisions with ships and levels of anthropogenic underwater noise disturbance that the species has not experienced for nearly two decades.
